# The Abuses of Ergot in Obstetrics*Read before the Buffalo Medical Club, Nov. 1, 1882.

**Published:** 1882-12

**Authors:** C. C. Frederick


### The Abuses of Ergot in Obstetrics.*

By C. C. Frederick, M. D.

Seventy-five years have passed since ergot was first brought to the notice of the profession in this country by Dr. Stearns, of Saratoga, in a letter to Dr. Ackerly concerning its use. Soon its power of producing strong uterine contractions became generally known, and as is the wont of Americans, generalizations were soon made from few data, and in ergot was foreseen the boon of every parturient woman, and a substitute for all instrumental interference. Accordingly, it became fashionable to give ergot indiscriminately in obstetric practice, and as a result the literature of this subject, for the first fifty years of its use, is full of instances of its enormous abuse. And yet, with all that has been written about this subject, there still are those practicing medicine, not only old but young men also, who are not aware of its dangers and who do not rightly appreciate the few indications for its use. Sir James Y. Simpson once said: "I believe few remedies have been more abused in practice."

In this connection it seems proper to determine, so far as possible, whether ergot has the power to originate uterine contractions. All admit that if the parturient effort has begun, ergot will intensify the pains, in fact produce tonic contraction. Such writers as Wood, Bartholow, Elliott, Cazeaux, Chevasse, Beck, Fordyce Barker, Pareira, Bayle, Waller, Holmes, and others, do not accord to it this power, or at least very seldom and only in exceptional cases. Mr. Wright, Bonjean, Chatard, Warner, Villeneuve, failed in every case to produce abortion in inferior animals. On the other hand, Chapman reports that in mammals he never failed to produce abortion in a short time.

* Read before the Buffalo Medical Club, Nov. 1, 1882.

Combs gave it to three pregnant bitches, abortion following. Diez, Oslere, Percy, and Laurent aborted guinea-pigs, cows, cats, rabbits and sows. Erskine always succeeded in aborting cats at various periods of pregnancy. M. Bodin reported an epidemic abortion of cows in 1842, near Trois Croix, France, attributed by him to ergotized grass. Gilman says, unqualifiedly, that ergot will abort the human female; Patterson of Glasgow, Prof. James, Mr. Heane, Drs. Dewers, Weicke, DuBois, Chatard, and others, add their testimony to the fact. Dr. Ramsbotham cites twenty-six instances of premature delivery produced by him with ergot alone, some labors following a single dose, others requiring the repetition of the dose thirty or forty times before pains began. Playfair says: "There is no doubt that ergot has the power of inducing uterine contractions."

From this evidence, I do not think we are justified in ascribing or denying to this remedy abortifacient power in all cases. The middle course seems the only reasonable ground, although there is no guide to the amount of confidence that can be reposed in it in any particular case.

If, in any instance, it does succeed in originating pains, to give it for this purpose, whether the premature delivery be with criminal intent or justifiable, is evidently wrong.

If given with criminal intent, and it succeeds, its worst effects can but be an inadequate retribution to the offending party for their transgression. When premature artificial labor is justifiable by reason of contracted or deformed pelvis, or excessive size of the fœtal head, there certainly are very cogent objections to its use, although the earlier obstetricians gave it for this purpose. Dr. Elliott said of such cases: "When it does act, it acts too much!" Allowing that full dilatation of the cervix should promptly follow the advent of the pains, thus ridding the mother of the probability of lacerated cervix, the powerful efforts of the uterus to force the child through the deformed or contracted canal are apt to make either a rupture of the uterus or kill the child, and possibly both. In a deformed pelvis there is apt to be a malpresentation. Rupture of the sac with ergot pains will, in all probability, take place before the position has been diagnosed, although since external palpation affords, with practice, so accurate a diagnosis of position, this objection is not so forcible as formerly. No doubt the accoucheur, to a great extent, is prevented from doing what he otherwise could do, to correct a malposition or assist nature in moulding the head to the maternal passages, and deliver the child and mother from their urgent danger. With the light of the past experience of others, I cannot understand how any man can so jeopardize the welfare of his patient.

It is a fact well known that ergot is very commonly given by many practitioners in abortion, from fear of hemorrhage. Abortions occurring during the third and fourth months of pregnancy are of two classes: one in which the sac is delivered entire; the other where the sac ruptures, the fluid and fœtus are expelled and the placenta and membranes are retained. The effort should always be to prevent rupture of the sac; therefore, ergot given before dilatation of the cervix would only rupture the sac by its powerful contractions. Dr. Nœggerath has seen abortions at the second and third month, when both the internal and external os were fully dilated, then ergot given caused a spasmodic contraction of the circular fibres of the inner os, with retention of the ovum. Even if the pains be feeble, ergot should not be given, except in severe hemorrhage, till the ovum has been so far expelled, entire, as not to allow the uterus to enclose it when it contracts. When hemorrhage occurs after rupture of the sac, ergot only serves to hold the placenta and membranes more securely in the uterine cavity, and is not sure or even probable to stop the hemorrhage. To remove the placental mass is the rational indication, and then give ergot, if necessary, to secure permanent contraction. Ergot is not to be relied upon, however, in all cases, hence the mistake should not be made to look to it for security from hemorrhage, when intra-uterine applications or the hot douche would be far more effective.

There are no indications for ergot in the first stage of labor, except in accidental hemorrhage and partial placenta praevia, in both of which Drs. Fordyce Barker and Lusk recommend its careful use. The tonic contractions which it produces, instead of the normal intermittent pains, tend to prevent the dilatation of the cervix. Suppose labor to begin, the os partially to dilate, pains cease and not recur again in several days, should ergot be given, artificial dilatation practiced and the woman delivered? Certainly not. I heard, not long since, of such a case, treated in the manner described. The results of ergot given in this stage may be rigidity of the cervix with subsequent laceration, rupture of the perinæum or uterus, or death of the child.

Ergot is not indicated in the second stage of labor, except for true uterine inertia, and then not so much to expel the child as to prevent post partum hemorrhage from uterine atony, because forceps are preferable for the delivery of the child. "Even under these circumstances," says Dr. Lusk, "it should not be given if there exist the slightest mechanical obstacle to delivery, or if the fœtal head be high in the pelvic cavity." In primiparæ, extra caution should be exercised. The fact of never having given birth to a child, together with the tension of the soft parts, contraindicate it. It is very necessary to determine if there be true uterine inertia. In hydramnii, the uterine walls being distended and too weak to rupture the membranes, pains often stop, ergot is not indicated, but removal of part of the liquor amnii. Labor may be retarded or pains stop from the resistance offered by an unyielding cervix. Ergot would probably rupture it. The uterus may be displaced, the head pressing against the sacrum; or a malpresentation not recognized; or a head not having undergone flexion; or there may be a distended bladder or rectum. In any of these the uterus is apt to tire after long-continued efforts to expel its contents. Here, certainly, a correct understanding of the causes of delay and the proper proceedings instituted for their relief, are more indicated than ergot. Then, after correcting the conditions which caused uterine exhaustion, if new stimulus is needed, Drs. Fordyce Barker and Albert H. Smith employ quinine in ten or fifteen grain doses, with excellent results, the effect being to stimulate the normal rythmical pains and prevent after-pains.

Sometimes pains maintain their normal rythm and force during the second stage, till the head reaches the floor of the pelvis. Under such circumstances, if the pains lose their expulsive force and are inefficient, a rigid perinæum is generally blamed for the delay. In the primipara, of course, much greater delay must be expected in softening the perinæum. This faulty action, as pointed out by Dr. Lusk, may be due to exhausted nerve-power, or to excessive uterine retraction. "In the latter case," says Dr. Lusk, "the faulty action results from the withdrawal upward and the consequent lessening of the intra-uterine pressure. * * * Hoffmeier found, in a number of instances, when the head rested on the pelvic floor, that the ring of Bandl, which was made out by palpation through the abdominal walls, was situated at from five to seven inches above the symphysis pubis, so that the contractile portion of the uterus covered not more than one-third of the fœtus. Under such circumstances, while the patient suffers from intense pain, the contractions of the partially-emptied uterus do not possess the force to overcome the resistance of a rigid perinæum." If no resistance is to be overcome other than that of the soft parts, the head having rotated in the pelvis, the assistance rendered by the abdominal muscles, combined with the method of expression recommended by Bidder, may suffice to the delivery. If rotation and flexion are not perfectly performed these forces may fail. "In such a case," to again quote Dr. Lusk, "the available remedies are ergot and forceps. Of these, the advantage of safety and celerity are all on the side of forceps. * * * When the tardy labor is due to tonic retraction, the use of ergot is calculated to aggravate the sources of delay. In other cases tonic retraction is the direct result of ergotic action."

When any indications arise for speedy delivery in either the first or the second stage, as in eclampsia, the favor of modern obstetricians is given to forceps, although Bayle has reported five cases of puerperal convulsions in which ergot successfully and rapidly accomplished the delivery. There is no doubt that many practitioners, either inexperienced or timid in the use of forceps, resort to ergot in expediting labor rather than use them. To choose between ergot and the unskillful use of forceps is certainly a difficult question, and is probably the one which perplexes many and determines their favoritism to ergot. Lusk says: "Many practitioners, however, who have observed that in practice ergot often acts, likewise, with speed and safety, accord to it a large measure of confidence, but along with these more fortunate experiences there is a shady aspect to be remembered."

When the anxiety of the accoucheur as to the safe delivery of the child is relieved, there still rises up before him the fearful spectre, the fear of post partum hemorrhage, and not until the placenta is delivered and the uterus in a state of tonic retraction and contraction does this phantom vanish. I believe it is quite common to administer a dose of ergot, just before the birth of the head or about that time, in order to secure good contractions at the delivery of the placenta, or to assist in its expulsion, especially if there has been a tendency to weak and tardy pains during the latter part of the second stage. It has been the experience of many, who have given ergot in the second stage or at its completion, to be troubled in the third stage with "hour-glass contractions," retained placenta, and more or less hemorrhage. Dr. Chevasse reports twenty-three cases of hour-glass contractions with retained placenta in his practice in seven years; Mr. Jukes had six in succession; Mr. Heane, Mr. Elkington, and Chevasse, Sr., report many. The more modern obstetricians frown upon this misuse of ergot, the mode of expression by Credé being entirely adequate to the successful completion of the third stage. Dr. Lusk expresses himself thus: "The tardy expulsion of the placenta, due to atony of the uterus, is of rare occurrence when the Credé method of expression is uniformly practiced." The same author very graphically gives the mechanism of hour-glass contraction and retained placenta: "After birth of the child, retraction is nature's safeguard against hemorrhage. As the result of the abuse of ergot, or in other cases from an abnormal adherence of the placenta, such an extreme degree of retraction may be reached before the completion of the third stage as to lead to the imprisonment of the placenta within the uterine cavity. In these cases, complete retraction in the body of the uterus is prevented by the presence of the placental mass. Below the latter, when no obstacle is opposed to the shortening of the muscular fibres, a constriction results. The constriction is most pronounced at the ring of Bandl." Thus ergot may defeat the very object for which it was given, and substitute for a short and easy third stage a long, tedious and painful effort to overcome the irregular contractions and secure the placenta. After expression of the placenta, ergot may be given to keep the uterus contracted; it is not to be entirely relied upon, however, in all cases. When should ergot be given for this purpose? Certainly not so early that its specific action can be exerted on the uterus before the completion of the third stage. Dr. Prescott, in a series of experiments, found its action to follow in from seven to twenty minutes after stomach administration, in the majority of cases in about ten minutes.

Dr. Beatty, of Dublin, pointed out to the profession, several years since, that post partum hemorrhage, due to uterine atony, occurring in a weak, delicate, lax-fibred, pale, spare woman, with weak but perhaps rapid pulse, and slow, feeble pains, can not be controlled by ergot, in fact, ergot increases the shock following the delivery, and if persisted in will kill her. Fordyce Barker has seen several deaths occurring under these circumstances, when a full dose of opium, combined with temporary measures to control the hemorrhage, would have been followed by tonic contractions of the uterus.

The dangers to which ergot may expose both mother and child demand our further attention. Dr. Trask, of White Plains, N. Y., in a paper on ruptured uterus, remarks that few practitioners are honest enough to report a rupture of the uterus following a dose of ergot which they have prescribed. For this reason very few journals ever have a report of the kind, and the statistics are necessarily very incomplete. Dr. Miller, in his obstetrics, refers to one medical journal, in 1841, which reported in one month two cases of rupture of the uterus from ergot, the names of persons and localities being suppressed.

Bandl has shown that ruptures of the uterus generally begin in the lower segment in the space between the ring bearing his name and the os externum, and that these ruptures follow a thinning of this segment, due to a retraction of the fundus and body of the uterus above the ring of Bandl. It readily appears, then, how ergot can be the primary cause of rupture of the uterus, since its characteristic action is tonic retraction of the body and fundus, as before stated.

Lacerations of the cervix and perinæum, it is readily seen, are far more apt to result from the tonic ergot pain than from the intermittent and slow dilating process of nature.

We certainly should not leave this subject and forget the little stranger who so often has laid down its life as an atonement for the misuse of ergot. Dr. Hosack wrote: "Ergot has been called, in some of the books, the pulvis ad partum; as regards the child it may, with equal truth, be denominated the pulvis ad mortum." This action upon the child has been theorized upon to the effect that the ergot was either carried to the child and produced a toxic effect; or, by pressure upon the head during the tonic pains, death resulted; or, the third and probably correct notion, that death is caused by compression of the placenta and cord, the child dying from asphyxia. Dr. Ramsbotham disproved the toxic effect on the child by observing that however much of the drug was taken by the mother, unless it produced its specific effect on the womb, the child did not suffer. McClintock and Hardy have observed that after its administration, in a majority of cases, a marked decrease is soon noticed in the pulsations of the fœtal heart falling to 100 or below, succeeded by irregular, then intermitting beats, and, after a variable period, the heart sounds became inaudible. Both of these observers, as well as Ramsbotham, found that if the child was not born within an hour, as a rule, after giving ergot, it would almost certainly be dead. Often they found it necessary to resort to forceps to save the child. It may be of interest to give some facts as observed by different obstetricians concerning this infant mortality from ergot: Davies, London, 10 born, 4 dead; Chevasse, 18 born, all dead; Patterson, 8 born, 3 dead; Mr. Chattò, in 420 labors, gave ergot 80 times; 422 children were born, 31 dead, 10 of the 80 and 21 of the remaining 340; one in eight with ergot died, without ergot one in seventeen died; McClintock and Hardy, 30 births, 20 dead; Dr. West, 69 born, 8 dead; Mr. Jukes, 6 born, 5 dead; Hosack, 3 born, all dead; Hardy, 48 born, 34 dead; Chatard, 37 born, 16 dead; Church, 7 born, 6 dead. I find that on the average, in all these cases reported above, one child out of every two and one-tenth died. Dr. Fordyce Barker reported, several years ago, that Dr. Stearns himself suffered so much in reputation from the infant mortality in practice by use of ergot that he was obliged to quit his field of labor and remove to New York City.

During the past twenty-five years I do not think ergot has been so grossly abused as in the half century preceding. There are those who think the reaction from that period of abuse is upon us, and that ergot is not used as often as indicated. It seems quite probable that the use of ergot in small doses to gently stimulate the tardy pains will come into more general vogue in the near future than now obtains. No doubt but that one abuse of ergot has been its exhibition in too large doses, thus getting its extreme effects. The subject of the use and abuse of ergot is one of great interest and merits the close study of every general practitioner, for every one does more or less obstetrics. It is doubly important, for in its right use or abuse two may be benefited or suffer.

